# Ovarian hormones through Wnt signalling regulate the growth of human and mouse ovarian cancer initiating lesions

**DOI:** 10.18632/oncotarget.11711

**Published:** 2016-08-30

**Authors:** Prathima B. Nagendra, Jyoti Goad, Sarah Nielsen, Loui Rassam, Janine M. Lombard, Pravin Nahar, Pradeep S. Tanwar

**Affiliations:** ^1^ Gynaecology Oncology Group, School of Biomedical Sciences and Pharmacy, University of Newcastle, Callaghan, New South Wales, Australia; ^2^ Hunter Cancer Biobank, University of Newcastle, Callaghan, New South Wales, Australia; ^3^ School of Medicine and Public Health, University of Newcastle, Callaghan, New South Wales, Australia; ^4^ Hunter Area Pathology Services, Calvary Mater Newcastle, Waratah, New South Wales, Australia; ^5^ Department of Medical Oncology, Gynaecology Oncology, Calvary Mater Newcastle, Waratah, New South Wales, Australia; ^6^ Gynaecology and Obstetrics, John Hunter Hospital, New Lambton Heights, New South Wales, Australia

**Keywords:** BRCA1/2, Wnt, ovarian cancer, hormone, fallopian tube

## Abstract

Ovarian cancer (OC) is the most deadly gynaecological disease largely because the majority of patients are asymptomatic and diagnosed at later stages when cancer has spread to other vital organs. Therefore, the initial stages of this disease are poorly characterised. Women with *BRCA1/2* mutations have a genetic predisposition for developing OC, but not all of these women develop the disease. Epidemiological findings show that lifestyle factors such as contraceptive use and pregnancy, a progesterone dominant state, decrease the risk of getting OC. How ovarian hormones modify the risk of OC is currently unclear. Our study identifies activated Wnt signalling to be a marker for precursor lesions of OC and successfully develops a mouse model that mimics the earliest events in pathogenesis of OC by constitutively activating βcatenin. Using this model and human OC cells, we show that oestrogen promotes and progesterone suppresses the growth of OC cells.

## INTRODUCTION

Ovarian cancer is the most deadly gynaecological cancer and the fourth leading cause of cancer death in women [1]. Every year, approximately 238,700 new cases are diagnosed and 151,900 deaths occur worldwide due to ovarian cancer [2]. Chemotherapy combined with debulking surgery is a standard treatment for ovarian cancer [3]. Ovarian cancer is one of the most chemosensitive solid malignancies and the initial response rate to standard treatment exceeds 80% [3]. However, most of these women will develop recurrent disease and eventually die because their cancer becomes resistant to chemotherapy, or has inherent chemo-resistance [3]. The 5-year survival rate of ovarian cancer patients has not significantly improved and is around 40% over the last 20 years [4], highlighting the need to understand the signalling pathways involved in the pathogenesis of this disease. This will allow us to identify novel therapeutic targets and thus develop novel means of treating this disease.

There are four major subtypes of ovarian cancer, namely, Clear cell, Endometrioid, Mucinous, and Serous [4]. The serous subtype is the most prevalent form of ovarian cancer and is responsible for 70-80% of ovarian cancer deaths [5]. Both ovary and fallopian tube are considered as the site of origin of ovarian cancer [6-9]. However, the majority of serous ovarian carcinomas are suspected to originate from the distal fallopian tube and then spread to the rest of peritoneal organs including the ovary [5]. Fallopian tube epithelium mainly consists of two cells types, secretory and ciliated cells. Secretory cells are believed to be the progenitors of serous ovarian cancer (SOC) [5, 9]. Extensive histopatholgical examination of fallopian tubes collected from patients that are predisposed to developing ovarian cancer revealed secretory cell expansions/outgrowths (SCE/SCOUTs) and/or serous tubal intraepithelial neoplasia/carcinoma (STINs/STICs) [10]. SCE/SCOUTs/STINs/STICs share many histological and molecular features with SOC. Engraftment of transformed human secretory cells into the peritoneum of immunocompromised mice leads to the development of tumours that are grossly, histologically, immunophenotypically, and genetically similar to SOC [11]. Additionally, secretory cell-specific genetic alterations in the *Brca1/2*, *Tp53*, and *Pten* genes using a Pax8-driven promoter causes development of tumors that are similar to human SOC [9]. Collectively, these findings suggest that deregulated signaling in the fallopian tube secretory cells induces SOC.

Various epidemiological and molecular studies have associated genetic and life style factors with the predisposition to developing ovarian cancer [5]. Germline mutations in breast and ovarian cancer susceptibility genes, *BRCA1* and *BRCA2*, significantly increase lifetime risk of developing ovarian cancer compared to the general population [5]. Patients with hereditary mutations in *BRCA1/2* genes have very high risk of developing SOC and are recommended to undergo risk-reducing salpingo-oophorectomy (RRSO) by age 40 [12]. Studies in large cohorts of women showed that breast-feeding, pregnancy/parity and combined oral contraceptive use significantly decreases, whereas, infertility and nulliparity increases their risk of developing ovarian cancer [13]. Combined oral contraceptive use is the most effective preventive measure against ovarian cancer and approximately 50% reduction in ovarian cancer risk occurs after 3-5 years of use [13, 14]. First full term pregnancy confers a 40% reduction in ovarian cancer risk, and every subsequent pregnancy after the first birth provides further risk reduction of 14% [15]. The protective effects of oral contraceptive use and pregnancy against ovarian cancer are postulated to occur due to high levels of progesterone hormone as combined oral contraceptive formulations with high progestin, synthetic progesterone agonists, are known to reduce ovarian cancer risk, whereas, low progestin and high oestrogen formulations have opposite effect [13]. How progesterone provides protection against developing ovarian cancer is currently unclear. In this study, we investigated the role of Wnt/βcatenin signalling in pathogenesis of SOC and showed the presence of active Wnt/βcatenin signalling in SCOUTs/STICs of human fallopian tubes. We have developed a mouse model by altering Wnt/βcatenin signalling that mimics the early stages of human SOC. Using human SOC cells and our mouse model, we have defined the role of oestrogen and progesterone in ovarian cancer development. Collectively, our data provides evidence that progesterone suppresses the growth of ovarian cancer initiating lesions and thereby provides protection against developing ovarian cancer.

## RESULTS

### Sustained activation of Wnt/βcatenin signalling in the precursor lesions of human SOC

**Figure 1:**
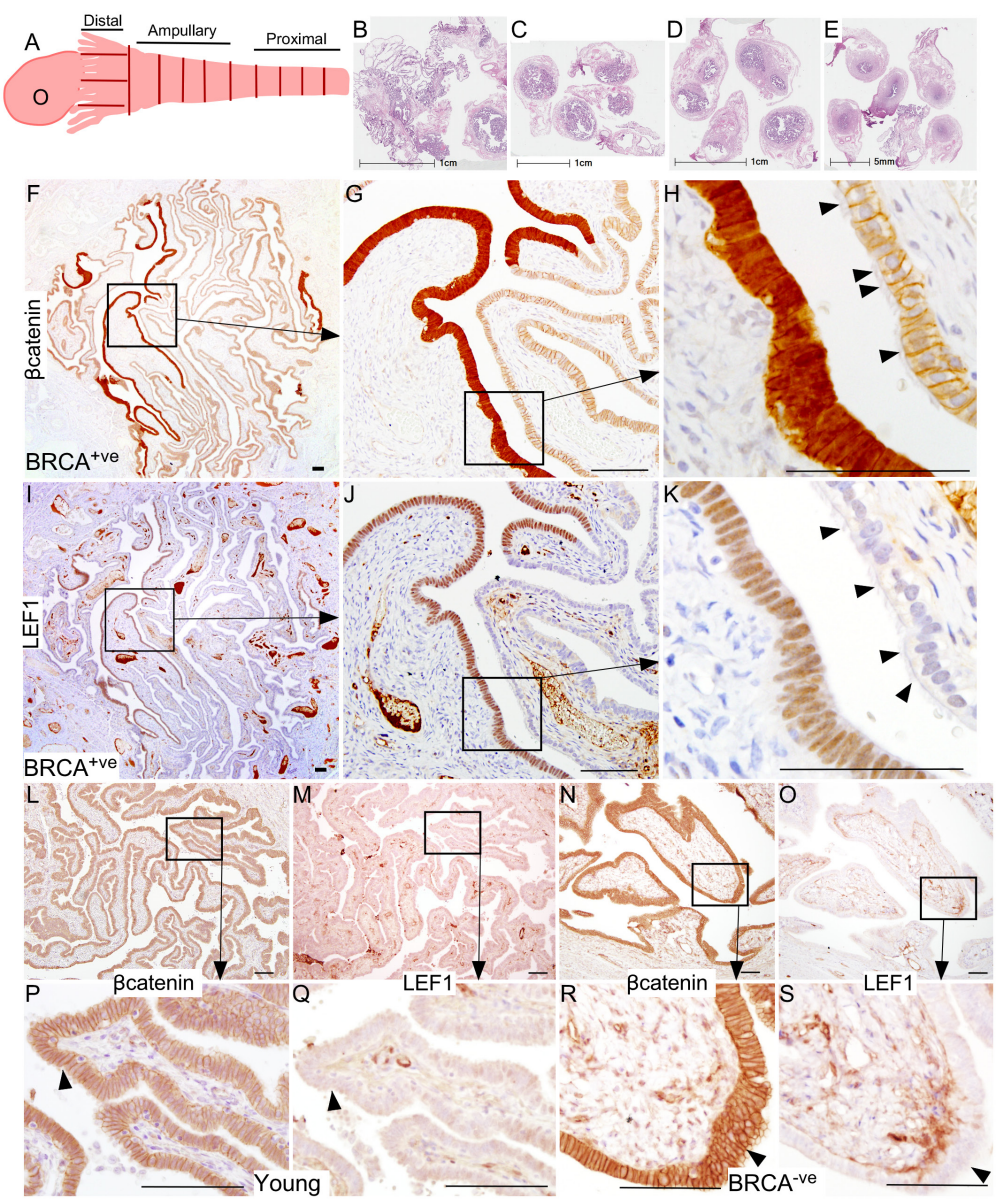
Hyperactive Wnt/βcatenin signalling is present in the Fallopian tube epithelium of *BRCA1/2* mutation positive women Whole fallopian tubes were collected from the *BRCA1/2* positive women who underwent risk-reducing salpingo-oophorectomy and were histologically examined for the presence of ovarian cancer precursor lesions using an established protocol described by Moorman *et al* [46] **A.** Representative tissue sections of the distal end (fimbriae, B), the middle region (ampulla, **C.** and **D.**) and the proximal end (isthmus, **E.**) are shown in panel **B.**-**E.** A typical ovarian cancer precursor lesion presented with nuclear/cytoplasmic (active form) βcatenin **F.**-**H.** and LEF1 **I.**-**K.** staining. Arrowheads in panel H and K are marking cilia. Representative sections from the fallopian tubes of young or *BRCA1/2* mutation negative women showed membranous/cytoplasmic βcatenin **L.**, **P.**, **N.**, **R.**; arrowhead) and absence of LEF1 staining **M.**, **Q.**, **O.**, **S.**; arrowhead). Bars: 100um.

Putative SOC precursor lesions (SCE/SCOUTs/STINs/STICs) are present in the fallopian tubes of patients at a high risk of developing ovarian cancer [10]. We collected whole fallopian tubes from 11 patients (*BRCA1/2* mutation positive or with a family history of breast or ovarian cancer) who underwent RRSO and performed extensive sectioning (∼3000 slides) to detect SOC precursor lesions under the supervision of a pathologist (L.R.) (Figure 1A-1E and Table 1). Wnt signalling plays a significant role in fallopian tube development and differentiation [16, 17]. Deregulated Wnt signalling is involved in carcinogenesis of various other organ systems [18]. To investigate if overactive Wnt signalling contributes to the pathogenesis of SOC, we performed immunohistochemical localization of βcatenin and LEF1, well-known targets of Wnt signalling [19], on serial sections of human fallopian tubes. We found patches of epithelial cells showing strong nuclear accumulation of βcatenin, which is indicative of active Wnt signalling (N: 11/11 patients; Figure 1F-1H). Staining of serial tissue sections revealed presence of LEF1 expression in same cells (N: 11/11 patients; Figure 1I-1K). These nuclear βcatenin and LEF1-positive epithelial patches were devoid of cilia, which were present on the normal looking adjacent epithelial cells (Figure 1H and 1K), suggesting that these epithelial patches are primarily consisting of secretory cells. As normal healthy women rarely undergo salpingo-oophorectomy, we were unable to procure the whole fallopian tubes from these females. We examined representative sections from the distal and the proximal end of fallopian tubes of young (*N* = 3; Age: 21-22 yrs; BRCA1/2 mutation status unknown) and adult (*N* = 3; Age: 33-65 yrs; *BRCA1/2* mutation negative) patients (Figure 1L-1S). We were unable to find epithelial lesions co-expressing nuclear βcatenin and LEF1 in these patients (Figure 1L-1S).

**Table 1:** Patient information

Patient number	Age (years)	*BRCA 1/2* mutation status
1	42	*BRCA1*
2	52	*BRCA2*
3	48	*BRCA1*
4	36	*BRCA1*
5	57	*BRCA2*
6	56	*BRCA2*
7	45	N.A. (history of breast cancer)
8	37	*BRCA1*
9	47	*BRCA2*
10	54	*BRCA2*
11	47	*BRCA2*
12	22	N.A.
13	21	N.A.
14	22	N.A.
15	63	Negative
16	55	Negative
17	33	Negative

N.A.: information not available or *BRCA1/2* mutation status was not determined.

**Figure 2:**
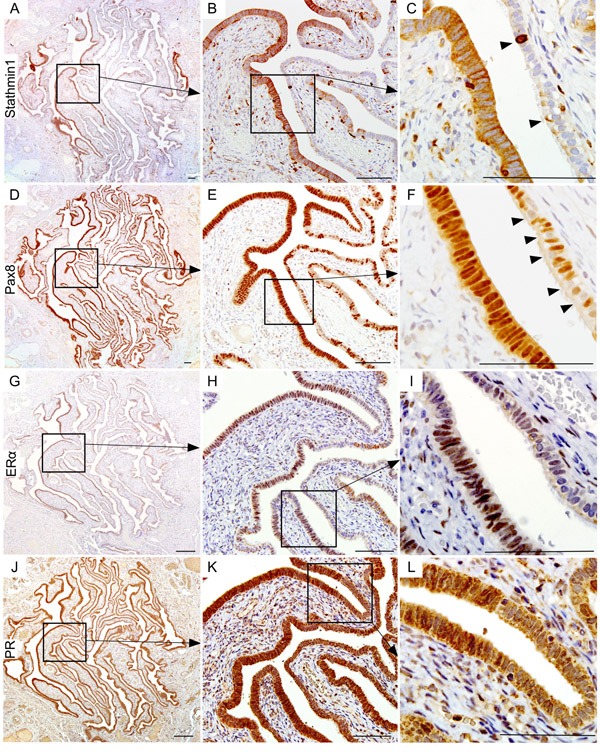
Wnt activation marks the human ovarian cancer precursor lesions Serial tissue sections of the *BRCA1/2* mutation positive fallopian tube epithelia with hyperactive Wnt signalling (nuclear βcatenin and LEF expression) also showed Stathmin1 **A.**, **B.** and **C.** and Pax8 **D.**, **E.** and **F.** expression, which are known markers of ovarian cancer precursor lesions. Arrowheads in panel C show intermittent positive cells for Stathmin1. Arrowheads in panel F are marking Pax8-negative ciliated cells, a typical feature of normal fallopian tube epithelia. These putative ovarian cancer precursor lesions also expressed ovarian hormone receptors, oestrogen receptor α (ERα; **G.**, **H.** and **I.**), and progesterone receptor (PR; **J.**, **K.** and **L.**). Bars: 100um.

**Figure 3:**
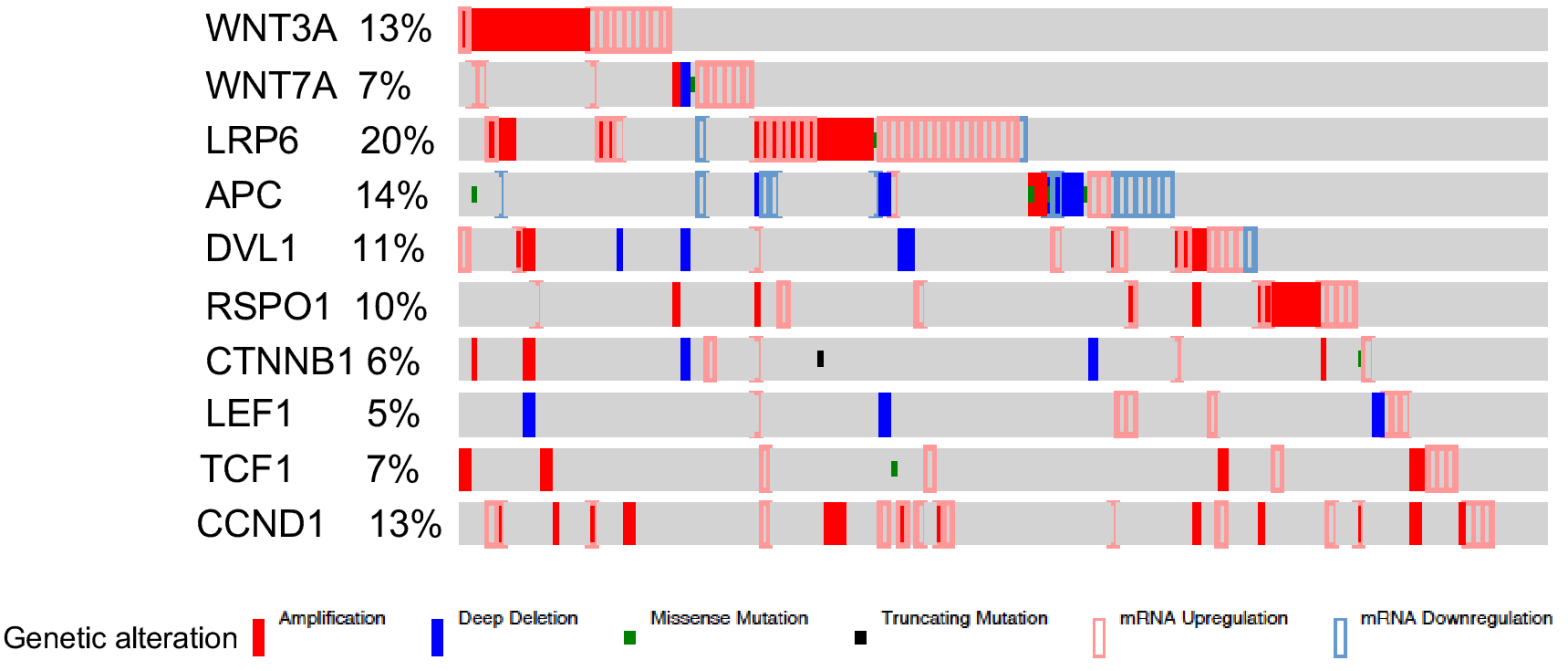
Alterations in the members of Wnt signalling pathway in human ovarian cancer patients Interrogation of the cancer genome atlas serous ovarian cancer dataset showed significant alterations (113 out of total 182 patients) in the Wnt pathway members.

To prove that the patches of epithelial cells presented with nuclear βcatenin and LEF1 expression represent SOC precursor lesions, we performed immunostaining for two well-established markers of SCE/SCOUTs/STINs/STICs and SOC, Stathmin 1 and Pax8 [9, 20, 21]. Fallopian tube epithelial cells with nuclear βcatenin and LEF1 expression were also positive for both stathmin 1 and Pax8 staining confirming their identity as SOC precursor lesions (Figure 2A-2F). These lesions also co-express oestrogen receptor α (ERα; Figure 2G-2I) and progesterone receptor (PR; Figure 2J-2L), suggesting that ovarian hormones might regulate their growth. Assessment of representative sections from the entire fallopian ducts of 11 patients with high risk of developing ovarian cancer revealed presence of cytoplasmic/nuclear βcatenin, LEF1, Pax8 and Stathmin 1-positive precursor lesions in all of the patients (SFigure 1). We observe variability in the number of lesions between different patients (SFigure 1). However, no correlation was observed between the number of lesions, patients' age, and the stage of menstrual cycle at the time of surgery. We also examined the Cancer Genome Atlas serous ovarian cancer database [22] and found genetic alterations in the Wnt pathway members in 62% (113/182) of the patients (Figure 3). In summary, these data showed that activation of Wnt/βcatenin signalling occurs in human SOC precursor lesions.

### Constitutive activation of Wnt/βcatenin signalling in mouse fallopian tube epithelium

**Figure 4:**
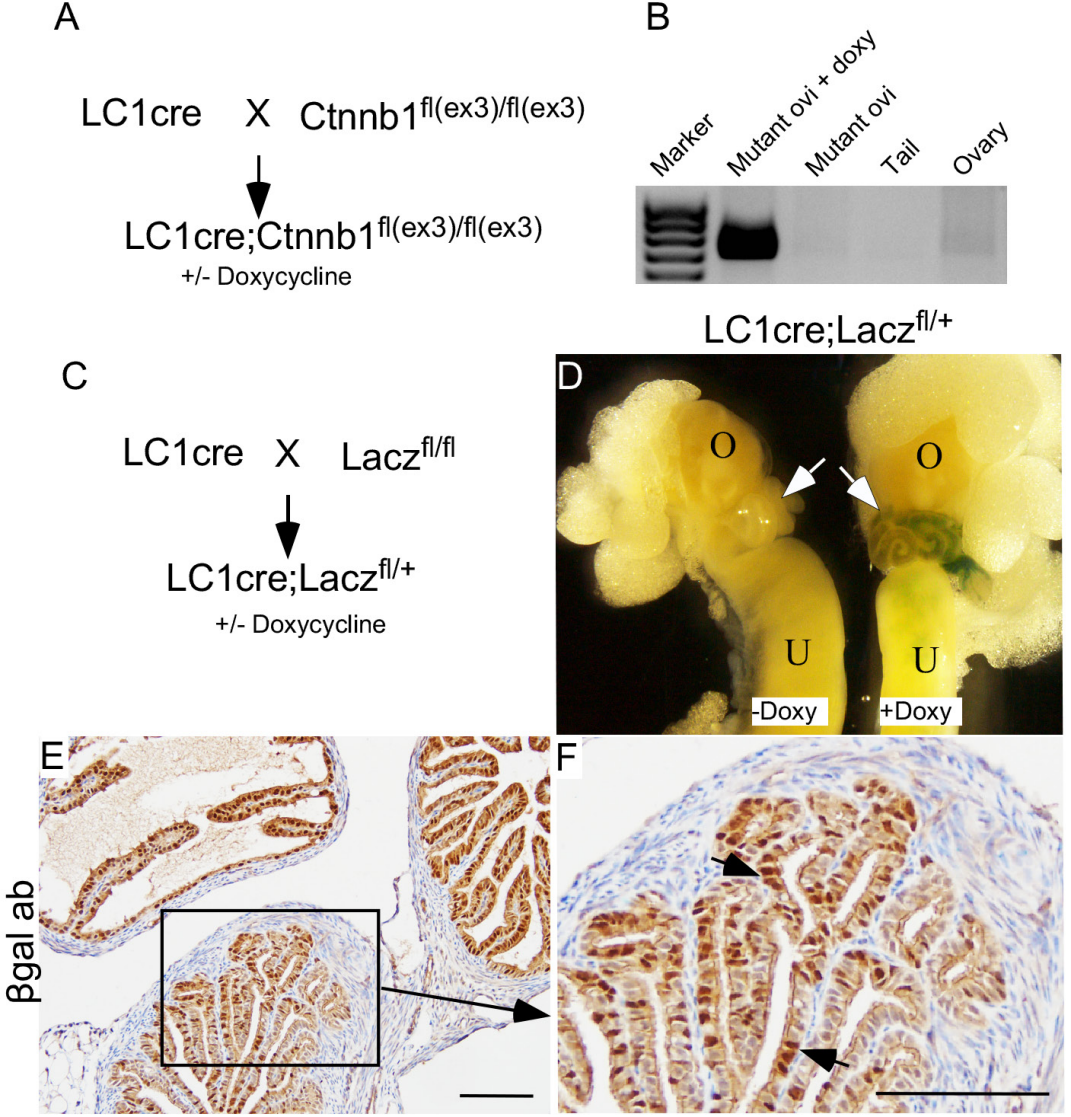
Constitutive activation of Wnt/βcatenin signalling in mouse fallopian tube secretory cells LC1cre mice were crossed with Ctnnb1^fl(ex3)/fl(ex3)^ to generate LC1 cre;Ctnnb1^fl(ex3)/fl(ex3)^ (βcatenin^ex3^cko; **A.**). Recombination PCR confirmed presence of floxed allele (band size: 0.7kb) in mutant oviduct but not in ovaries **B.** Mouse tails were used as negative controls **B.** Breeding strategy used for developing LC1cre driven lacZ reporter mouse model **C.** The gross image of the female reproductive tract isolated from mice treated with and without doxycycline showed recombination specifically in the fallopian tubes (arrows) of doxycycline treated LC1cre; Lacz^fl/+^ mice, but not in the ovary **D.** Fallopian tube section from LC1cre; Lacz^fl/+^ mouse stained for βgalactosidase showed secretory cell specific expression (arrows; **E.** and **F.**). Bars: 100um.

Studies using human and animal models have provided evidence that uncontrolled growth of fallopian tube secretory cells is responsible for the pathogenesis of SOC [9]. Pax8 is a bona fide marker of both human and mouse secretory cells [9]. To test if hyperactive Wnt signalling in secretory cells is responsible for the development of SCE/SCOUTs/STINs/STICs, we developed a mouse model (βcatenin^ex3^cko) in which constitutively active form of βcatenin is expressed under the control of a Pax8-driven reverse tetracycline-controlled transactivator combined with a tetracycline-responsive Cre recombinase (*Pax8^rtta^Tetocre or LC1cre*; Figure 4A). βcatenin^ex3^cko mice were given doxycycline in their drinking water (0.2mg/ml, adlibitum) to induce recombination of the flox alleles. Using primers directed to detect the recombined knock-in alleles of the *βcatenin*, we showed that recombination of the *βcatenin^ex3^* allele occurs in fallopian tube (Figure 4B). Mouse Tail DNA was used as a negative control as Pax8, and thus Cre expression is absent in this tissue [9] (Figure 4B). To confirm that *Pax8* promoter driven *Cre recombinase* is specifically targeting the flox alleles in the mouse fallopian tube secretory cells, we developed another mouse model (LC1cre;Lacz^fl/+^) by crossing LC1cre mice with Lacz reporter mice (Lacz^fl/fl^; Figure 4C). Whole mount βgalactosidase (βgal) staining of the female reproductive tracts collected from LC1cre;Lacz^fl/+^ mice treated with or without doxycycline showed Lacz expression in fallopian tubes but not in the ovaries of mutant mice that were exposed to doxycycline (Figure 4D). No Lacz expression was observed in untreated LC1cre;Lacz^fl/+^ mice, which was used as negative control (Figure 4D). Using an antibody against βgal, we confirmed that LC1cre driven recombination was limited to the fallopian tube secretory cells of LC1cre;Lacz^fl/+^ mice (Figure 4E and 4F).

### Sustained activation of βcatenin leads to abnormal outgrowths of secretory cells

**Figure 5:**
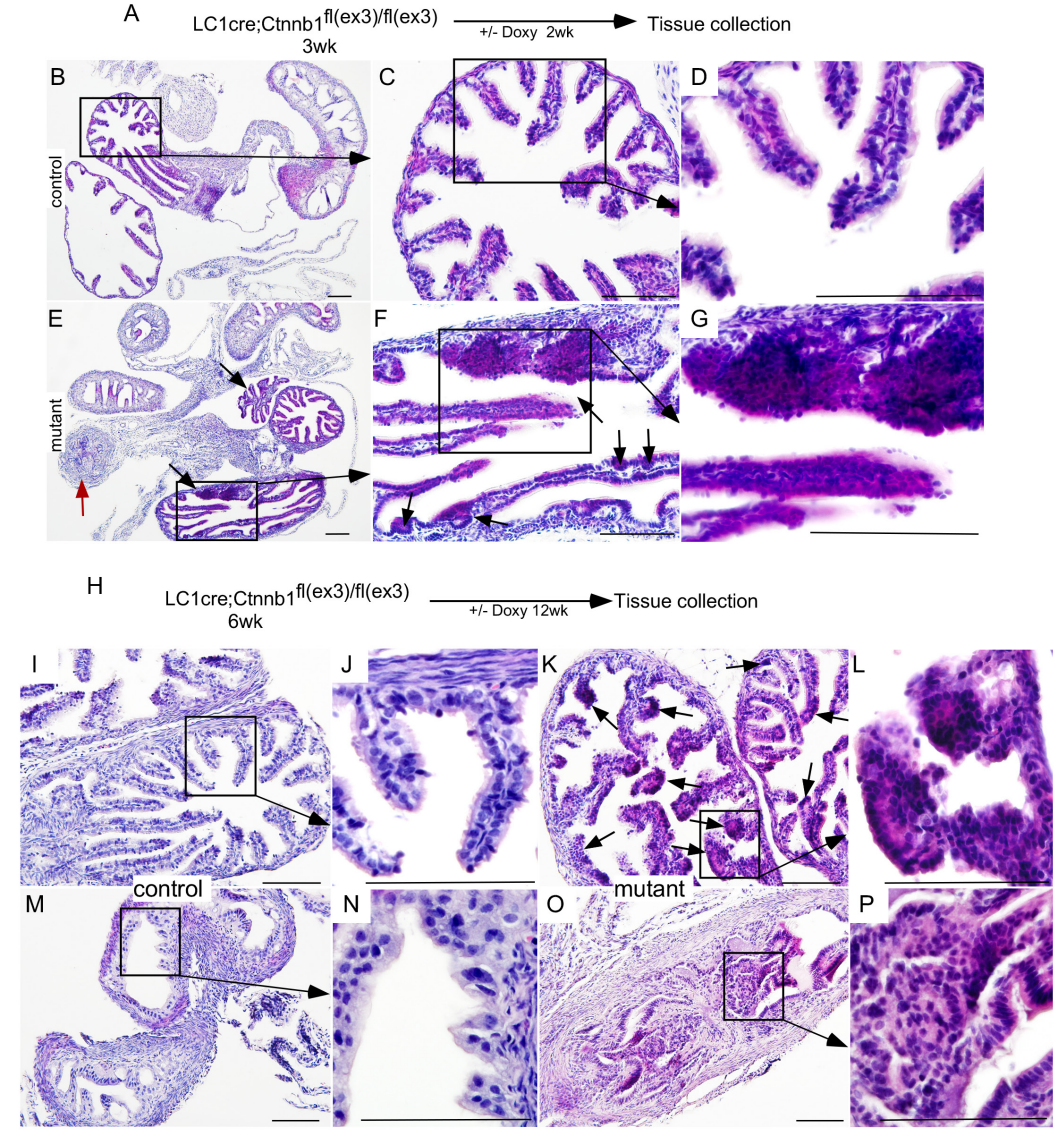
Sustained Wnt/βcatenin signalling in the fallopian tube secretary cells leads to abnormal epithelial growths similar to human SCOUT/STIC Short-term (2wk) doxycycline treatment of βcatenin^ex3^cko mice **A.** H&E stained Fallopian tube sections from control and short-term doxycycline treated mutant mice **B.**-**G.** The mutant fallopian tubes showed epithelial hyperplasia (black arrows) and occluded lumen (red arrow; **E.**-**G.** Long-term treatment with doxycycline of mutant mice resulted in wide spread epithelial dysplasia, focal growths (arrows, **K.** and **L.**), and blockage of the fallopian tube lumen (**O.** and **P.**). Morphologically normal fallopian tubes from control mice (**I.**, **J.**, **M.** and **N.**). Bars: 100um.

To determine the effects of overactive Wnt/βcatenin signalling, we collected female reproductive tracts from βcatenin^ex3^cko mice treated with doxycycline for 2wks (short-term; Figure 5A). Compared to controls (Figure 5B-5D), histological examination of mutant mice showed nodular and focal expansion of epithelial cells in the distal fallopian tubes (Figure 5E-5G; *N* = 5/5). Abnormal growth of epithelial cells was also observed in the proximal fallopian tubes (Figure 5E, marked with a red arrow). To test if long-term administration of doxycycline would increase the severity of mutant mice phenotype, we examined fallopian tubes collected from βcatenin^ex3^cko mice exposed to doxycycline for 12wks and found intraepithelial tumorous growth similar to human STIC in both the distal and the proximal fallopian tubes (Figure 5K, 5L, 5O and 5P; *N* = 7/7). No such growths were present in control mice (Figure 5I, 5J, 5M and 5N; *N* = 5/5).

**Figure 6:**
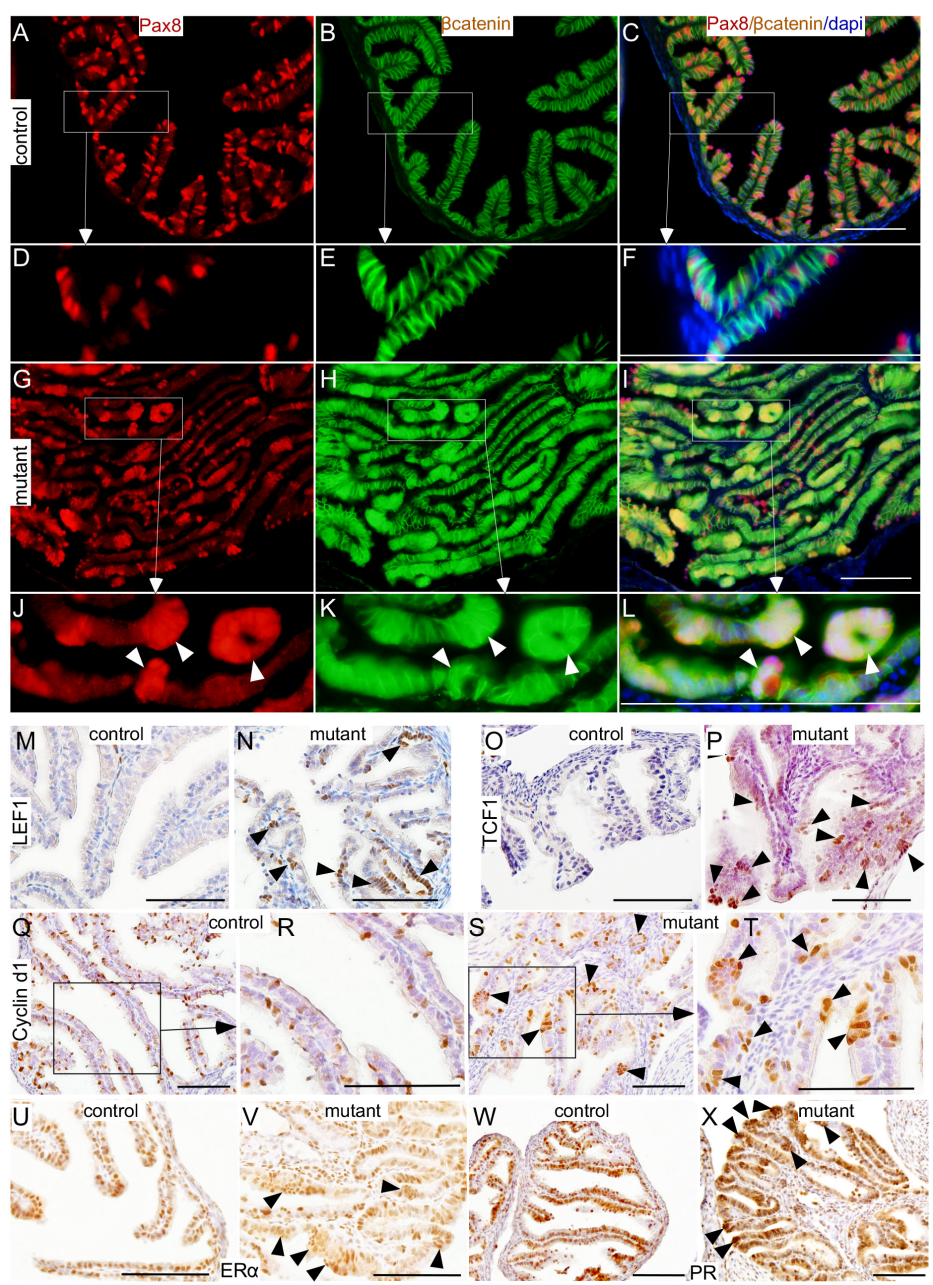
Histopathological analysis of abnormal changes in the mutant fallopian tube epithelium Pax8 and βcatenin expression in fallopian tubes collected from control mice **A.**-**F.** Nuclear/cytoplasmic accumulation of βcatenin in the Pax8-positive epithelial lesions (arrowheads) present in βcatenin^ex3^cko fallopian tubes **G.**-**L.** Expression of LEF1 (**M.** and **N.**), TCF1 (**O.** and **P.**), cyclin d1 (**Q.**-**T.**), ERα (**U.** and **V.**), and PR (**W.** and **X.**) in control and mutant fallopian tubes. Arrowheads mark the epithelial lesions that are positive for the probed proteins. Bars: 100um.

Studies in fallopian tubes collected from asymptomatic women with germ line mutations in *BRCA1/2* genes have suggested stepwise progression to SOC [10]. The earliest lesions identified in these patients' fallopian tubes are SCEs followed by SCOUTs and then STINs/STICs [10]. To confirm that epithelial growths in mutant mice originated from the secretory cells and phenocopy human SOC precursor lesions, we performed colocalization of βcatenin and Pax8 (Figure 6A-6L). In controls, a discrete pattern of Pax8 expression was observed in the distal fallopian tubes where positive secretory cells were interspersed between negative ciliated cells (Figure 6A-6F). In mutants, abnormal outgrowths present in the fallopian tube epithelium were Pax8-positive confirming their identity as secretory cells (Figure 6G and 6J). Examination of βcatenin expression revealed nuclear accumulation of βcatenin in these Pax8-positive lesions (Figure 6H and 6K). In contrast, mainly membranous βcatenin localization was seen in fallopian tubes of control mice (Figure 6B and 6E).

LEF1, TCF1 and Cyclin d1 are well known downstream targets of Wnt/βcatenin signalling [18, 19]. Examination of their expressions showed increased LEF1, TCF1 and Cyclin d1-positive cells in mutant fallopian tube epithelial cells compared to controls (Figure 6M-6T), which further provide evidence for hyperactivation of this pathway in mutant mice compared to controls. Collectively, these results showed that constitutive activation of Wnt/βcatenin signalling leads to abnormal secretory cell outgrowths that are similar to those observed in human patients who are predisposed to developing ovarian cancer.

### Oestrogen promotes and progesterone suppresses SOC precursor lesions growth

Studies in human patients suggest that hormones play a key role in the development of ovarian cancer [15]. Differences in the rate of fallopian tube epithelial cell proliferation rate are observed between the follicular and the luteal phase of the ovarian cycle suggesting that oestrogen and progesterone signalling is an important regulator of the fallopian tube functions [23]. Analysis of oestrogen and progesterone receptor (ER and PR) showed normal expression in both the control and the mutant fallopian tube epithelial cells (Figure 6U-6X). Interestingly, both ER and PR were also present in abnormal epithelial outgrowths of the mutant fallopian tubes suggesting that their development might be regulated by the changes in the levels of ovarian hormones (Figure 6V and 6X).

**Figure 7:**
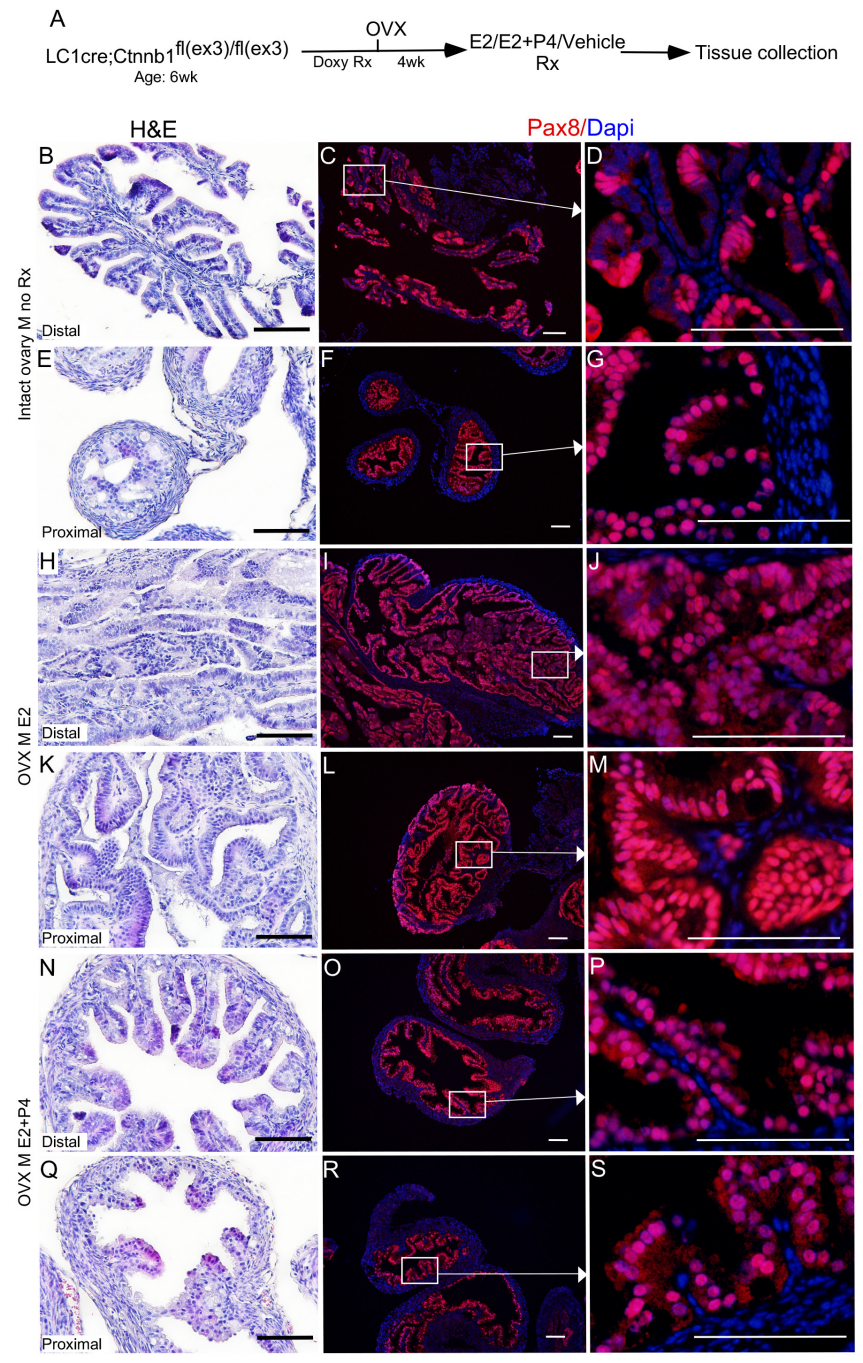
Oestrogen promotes and progesterone suppresses the growth of precancerous lesions in the mutant fallopian tube epithelium βcatenin^ex3^ckomice were ovariectomised to remove endogenous hormones and were subjected to doxycycline and hormonal treatments sequentially, as shown in **A.** Hyperplasia in the fallopian tube epithelium of mutant mice belonging to the control group **B.**-**D.** The Pax8 expression showed focal expansions in the fallopian tube of βcatenin^ex3^ckomice **C.** and **D.** The cohort subjected to oestrogen treatment developed intraepithelial carcinoma in both the distal **H.** and proximal **K.** fallopian tube. Panel **I.**, **J.**, **L.** and **M.** shows the secretory cell positive epithelial outgrowths. Mice treated with both oestrogen and progesterone displayed reduced abnormal epithelial growths **N.**-**S.** and were comparable to controls **B.**-**G.** Bars: 100um.

**Figure 8:**
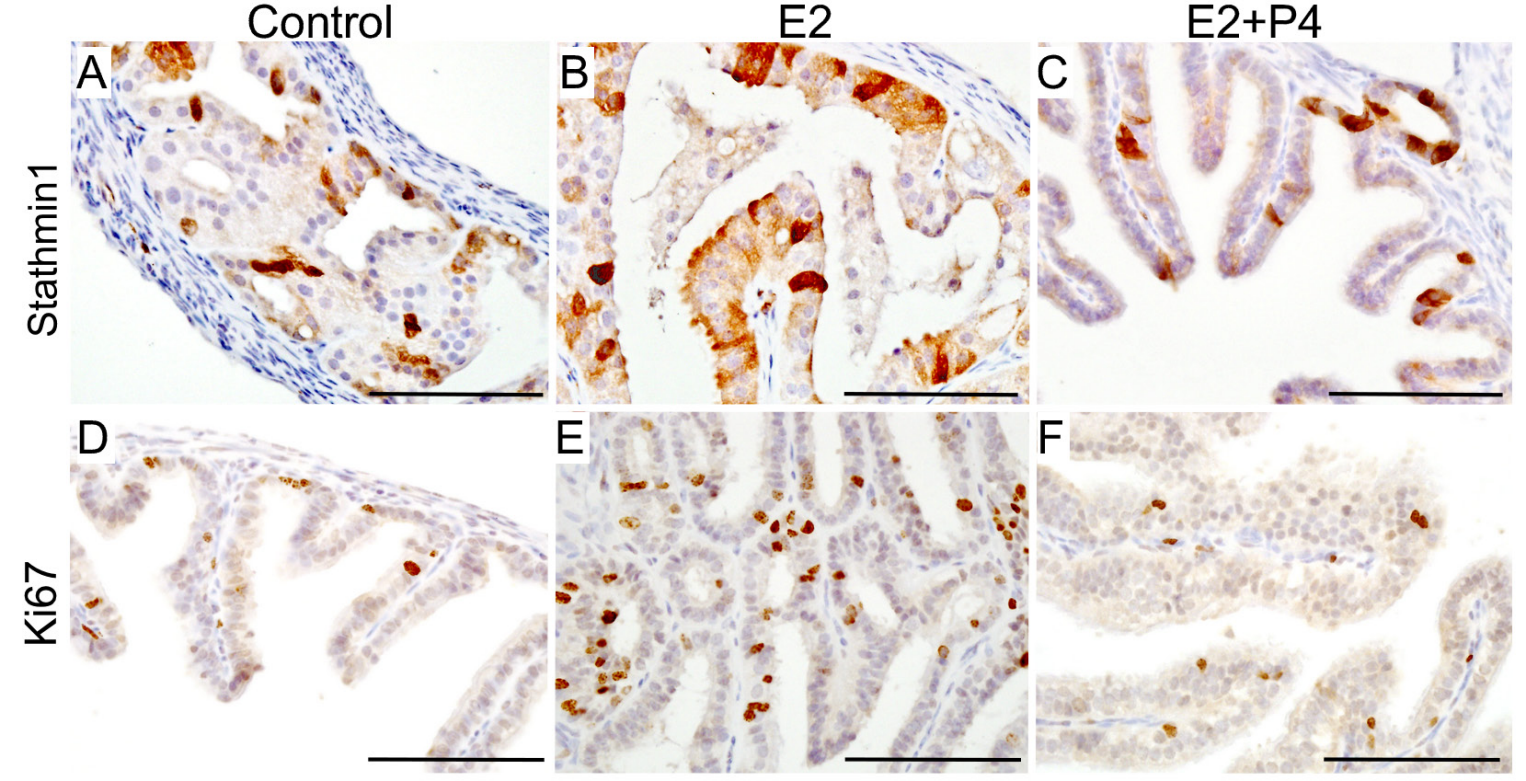
Expression of human ovarian cancer markers in the tumorous lesions observed in the fallopian tube of mutant mice Expression of Stathmin 1, a well established marker of serous ovarian cancer, in the fallopian tube epithelial growths of control (mutant mice with intact ovaries; **A.**), oestrogen treated group (E2; **B.**) and oestrogen plus progesterone treated group (E2+P4; **C.**). Ki67, a marker of proliferating cells, showed an increase in the rate of proliferation of the fallopian tube epithelial cells in E2 treated group **E.** compared to control **D.** and E2+P4 group **F.** Bars: 100um.

To test if oestrogen and progesterone supplementation influences the initiation of SOC, we surgically removed both ovaries from βcatenin^ex3^cko mice (*N* = 10, age: 6wks) and allowed the mice to rest for 14 days to remove any trace of circulating ovarian hormones (Figure 7). After the resting period, oestrogen (0.72 mg/90 day release) or oestrogen and progesterone (0.72 mg + 100mg/90 day release) pellets were subcutaneously placed in these mice (*N* = 5/group). For controls, sham surgeries were performed on βcatenin^ex3^cko mice of the same age and tissues were collected at the same time with two other groups (*N* = 5; Figure 7A). Histological examination and Pax8 immunolocalization showed abnormal enlargement and intraepithelial cancerous growth in both the distal and the proximal end of fallopian tubes of the oestrogen treated group (*N* = 5/5; Figure 7H-7M). These epithelial tumours invaded and occluded the lumen of the mutant fallopian tubes (*N* = 5/5; Figure 7H and 7K). In contrast, oestrogen and progesterone treated mice fallopian tubes were comparable to the control group (*N* = 5/5; Figure 7N-7S and 7B-7G). These tumours in oestrogen treated group were highly invasive as epithelial cells were present in the muscular and serosal layers of fallopian tubes (*N*= 5/5; SFigure 2). However, no evidence of metastasis was observed in any of these mutant mice, suggesting that additional genetic or molecular events are required for the metastatic spread of SOC cells. These murine tumours expressed markers of early and late human SOC, Pax8 and Stathmin 1 (Figure 7 and 8A-8C). Immunostaining for Ki67 depicted increase in proliferating cells in oestrogen treated group compared to oestrogen and progesterone and control (mutant mice with intact ovaries) mice (Figure 8D-8F). In summary, these experiments have shown that ovarian hormones are key regulators of SOC growth and provide an explanation for the effectiveness of combined oral contraception and pregnancy, high progesterone conditions, in suppressing the development of SOC.

### Ovarian hormones affect Wnt/βcatenin signalling for regulating the growth of SOC

**Figure 9:**
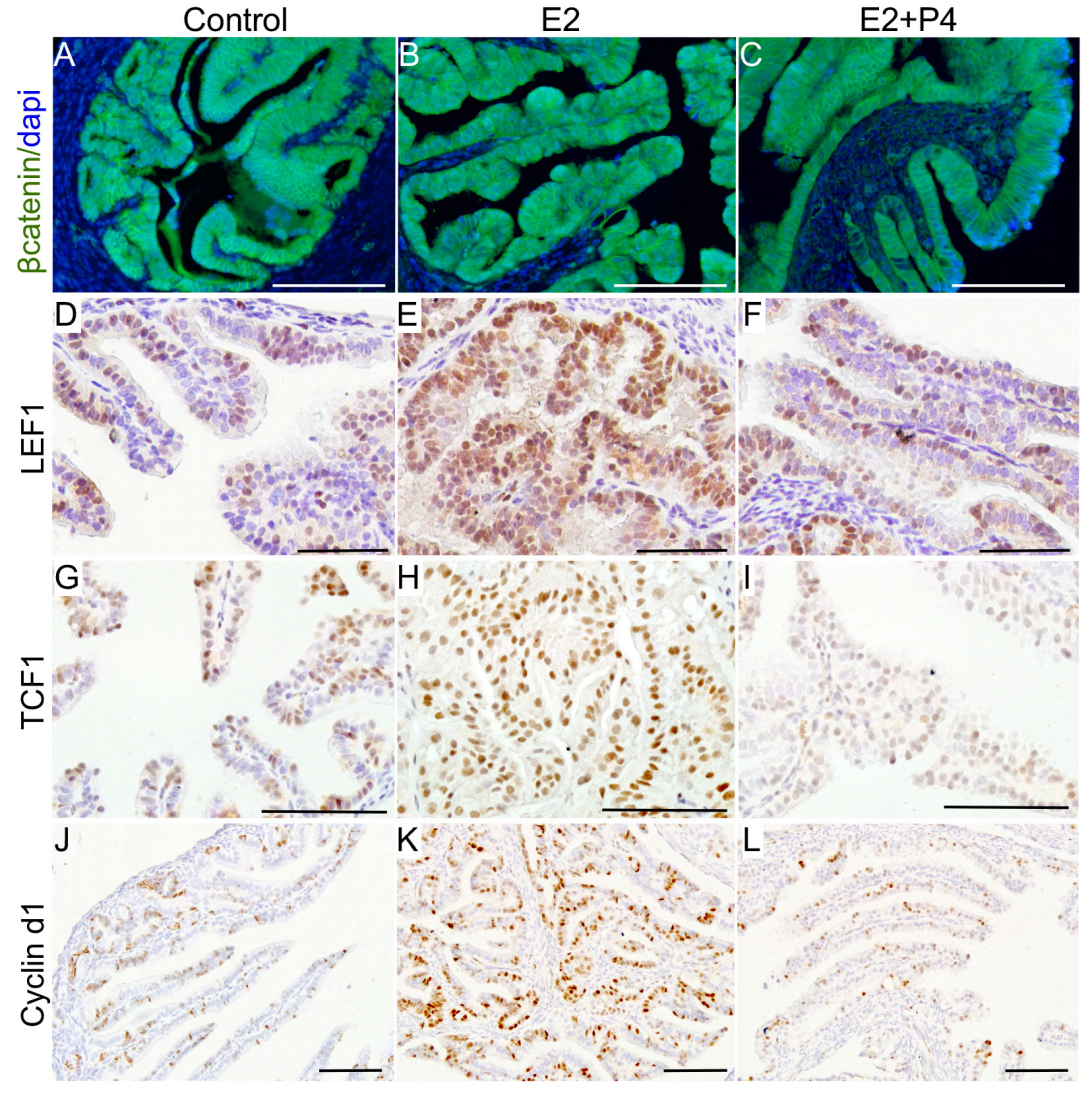
Ovarian hormones modulate Wnt/βcatenin signalling to affect the growth of precancerous lesions Increased expression of βcatenin and its downstream targets, LEF1, TCF1 and Cyclin d1, in the fallopian tubes of oestrogen (E2; **B.**, **E.**, **H.** and **K.**) treated group compared control (mutant mice with intact ovaries; **A.**, **D.**, **G.** and **J.**) and oestrogen plus progesterone (E2+P4; **C.**, **F.**, **I.** and **L.**) group. Bars: 100um.

To examine if changes in ovarian hormone levels affect Wnt signalling, we analysed the expression of βcatenin and its downstream targets (LEF1, TCF1 and Cyclin d1) in the fallopian tube samples collected from the βcatenin^ex3^cko mice treated with oestrogen, oestrogen and progesterone, and control group. The assessment of βcatenin expression revealed increase in nuclear/cytoplasmic localization of this protein, indicative of active Wnt signalling, in oestrogen treated group compared to controls (Figure 9A-9C). Co-treatment with progesterone mitigated the effects of oestrogen on βcatenin localization and reduced its expression compared to the oestrogen treated and control group (Figure 9A-9C). We have previously established that cells with active βcatenin signalling show increased nuclear expression of LEF1, TCF1 and Cyclin d1 [19]. Examination of LEF1, TCF1 and Cyclin d1 expression showed significant increase in their expression in oestrogen treated group compared to controls (Figure 9D-9L). Interestingly, co-treatment with progesterone reduced the expression of all these three markers in the mutant fallopian tube epithelial cells compared to the oestrogen treated group (Figure 9D-9L). These findings suggest that the inhibitory effect of progesterone treatment on the initiation of SOC might involve suppression of Wnt signalling.

**Figure 10:**
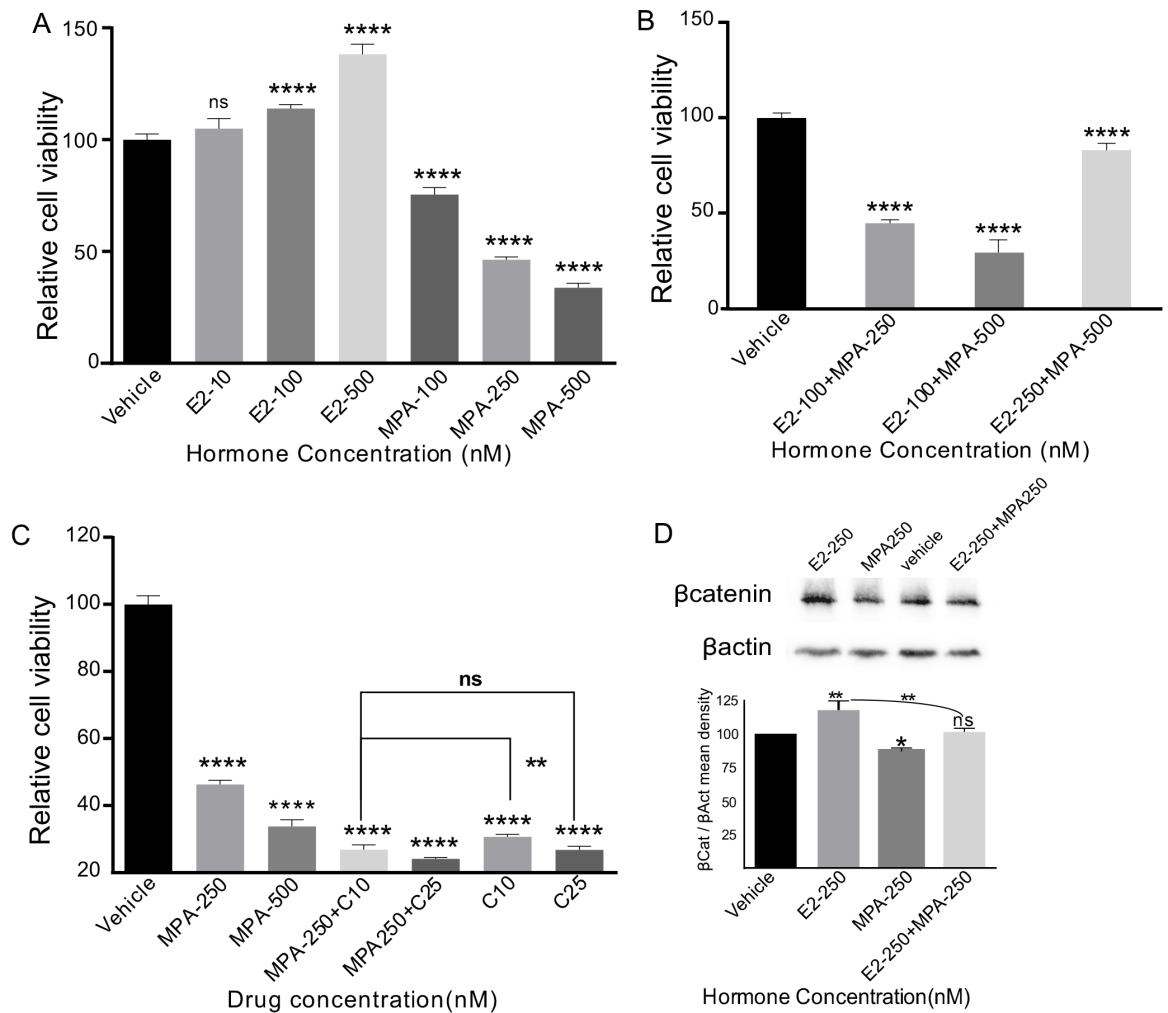
Oestrogen promotes and progesterone suppresses the growth of human serous ovarian cancer cells PE01 cells treated with incremental doses of 17-β-oestradiol (E2) showing increase in cellular viability in a dose dependent fashion **A.** Medroxyprogesterone acetate (MPA) treatment had an opposite effect **A.** Co-treatment of PEO1 cells with E2 and MPA resulted in suppression of the growth promoting effect of oestrogen by MPA **B.** However, surplus concentrations of 17-β-oestradiol with lower concentrations of MPA further increased the cellular viability of PEO1 cells **B.** Cellular viability of PE-01 cells treated with incremental doses of MPA and MPA+Carboplatin **C.** E2 and/or MPA treatment affects the βcatenin levels **D.** βactin was used as a loading control **D.** The data shown are representative of three individual Western blot analyses. *p* value *panel A-C* **P = 0.0018, ****P = 0.0001, ns P = 0.1053 (panel A), P = 0.9998 (panel C), P = 0.4771, panel D, *P = 0.0115, **P = 0.0020, ns P = 0.9534, One-Way Anova test.

To assess whether ovarian hormones regulate the growth of SOC, we treated PEO1 cells, a well-characterized SOC cell line that is known to express oestrogen and progesterone receptors [24], with varying doses of oestradiol (100nM, 250nM and 500nM) and/or medroxyprogesterone acetate (MPA; 100nM, 250nM and 500nM). Drug doses were determined on the basis of previous studies [25, 26]. Consistent with our observations in the mouse model, oestradiol treatment significantly increased PE-01 cell viability in a dose dependent manner (Figure 10A). The SOC cell viability was decreased with incremental doses of MPA alone (Figure 10A). Co-treatment with MPA mitigated the effect of oestradiol (Figure 10B). Furthering this, the current standard of care chemotherapeutic drug, carboplatin was combined with incremental doses of MPA and our data showed that MPA treatment enhances the efficacy of carboplatin, the higher doses of MPA being as efficient as the lower doses of carboplatin (Figure 10C). As expected, no response to oestradiol treatment was observed in another ovarian cancer cell line, COV362, which lacks the expression of oestrogen and progesterone receptors (data not shown).

To understand whether ovarian hormones affect Wnt signalling in ovarian cancer cells, PE01 cells were plated in six well plates and were treated with either oestradiol (500nM) or MPA (500nM) or oestradiol (500nM) + MPA (500nM) or DMSO. Compared to controls, treatment with oestradiol increased the level of βcatenin protein (Figure 10D), whereas, co-treatment with MPA had an opposite effect (Figure 10D). These results indicate that ovarian hormones regulate the growth of human SOC by modulating Wnt/βcatenin signalling.

## DISCUSSION

The fallopian tubes are the tubal organs that physically connect the ovaries to the uterus. These organs are the sites for egg sperm fusion and provide an appropriate environment for early embryonic development and transport. Studies from human patients with increased risk (mainly with germ line mutations in the *BRCA1/2* genes) to ovarian cancer have provided evidence for the fallopian tube epithelium being one of the sites of origin for SOC [5, 27]. This is further supported by the findings that the surgical removal of fallopian tubes (bilateral salpingectomy) significantly lowers the risk of developing ovarian cancer compared to women with no surgery [28]. Similar to the breasts and the ovaries, fallopian tubes are affected by changes in hormone levels during the oestrus cycle. Examination of fallopian tubes from women with or without *BRCA1/2* mutations revealed increased proliferation of epithelial cells during the follicular phase, an oestrogen dominant phase, compared to the luteal phase of the oestrous cycle, a progesterone dominant phase [23]. Epidemiological studies in human patients with hereditary predisposition to ovarian cancer have indicated that increased number of ovulatory cycles confers high risk of developing ovarian cancer [29]. It has been hypothesised that with every ovulatory cycle, the fallopian tube epithelial cells are bathed with oestrogen rich follicular fluid and that the unopposed mitogenic effects of oestrogen on these epithelial cells lead to neoplastic growth [30]. This hypothesis is supported by *in vitro* findings showing that exposure of the fallopian tube epithelial cells to follicular fluid induces DNA damage and increases proliferation mimicking the early events leading to SOC [31].

Studies examining cancer risk in *BRCA1/2* mutation positive women have shown that approximately 50% of these women develop breast or ovarian cancer by the age of 70 years, but approximately 50% of these patients even in their late 80′s don't develop these cancers [32], suggesting that in addition to genetic changes, other factors, such as lifestyle, influence the risk of developing reproductive cancers [13, 33]. A large study examining 31,658 catholic nuns showed that these women are more likely to die from reproductive cancers such as breast, ovarian and uterine cancers compared to the general population [33]. This study suggests that uninterrupted exposure to high levels of ovarian hormones for long durations might contribute to the pathogenesis of reproductive cancers. In support, a long-term follow up study of 17,032 women revealed that oral contraceptive use significantly lowers the risk of ovarian cancer without affecting the risk of breast cancer [34]. Breast-feeding, oral contraceptive use, and parity are associated with decreased risk of developing ovarian cancer in *BRCA1/2* mutation carriers [29]. High progestin oral contraceptive formulations provide greater protection compared to the low progestin formulations [13]. Similarly, twin pregnancies, a high progesterone physiological condition, provide better protection against ovarian cancer than singleton pregnancies [13]. These findings suggest progesterone and its mimetic provide protection against ovarian cancer. In contrast, oestrogen dominant conditions such as nulliparity and oestrogen-only hormone replacement therapy have opposite effects. How these ovarian hormones affect the development of ovarian cancer is currently unclear. In our study, we have shown that human SOC precursor lesions express oestrogen and progesterone receptors suggesting that these ovarian hormones affect their growth (Figure 1). Treatment of a SOC mouse model showed that oestrogen increases and progesterone decreases the growth of SOC precursor lesions (Figure 7). Collectively, these findings provide a molecular explanation to the epidemiological observations that high progesterone conditions provide protection against ovarian cancer.

Studies from our lab and others have shown that Wnt/βcatenin signalling plays a major role in female reproductive tract development and its deregulation leads to various reproductive tract diseases including ovarian cancer [35-37]. Mouse knockout studies have revealed that conditional loss of *βcatenin* results in shortening and defective coiling of the fallopian tubes, partially due to the lack of proliferation of epithelial cells [16, 36], indicating that normal Wnt/βcatenin signalling is essential for the fallopian tube development. Recently, two different studies in mice have identified that leucine-rich repeat-containing heterotrimeric guanine nucleotide-binding protein-coupled receptor 5 (LGR5), a member of the Wnt signalling receptor complex, marks stem/progenitor population of the ovarian surface epithelium and the distal fallopian tube [17, 38]. In this study, we have discovered that active Wnt/βcatenin signalling marks the precursor lesions of SOC in human fallopian tubes and the sustained activation of this signalling pathways leads to the development of similar lesions in mouse fallopian tubes (Figure 1-4). These findings raise an exciting possibility that ovarian cancer is a disease caused by the rogue stem/progenitor cells that have acquired changes culminating in overactive Wnt signalling. Similar to intestinal cancer development model [39], sustained activation of the Wnt pathway might cause defects in differentiation of the fallopian tube stem/progenitor cells leading to the abnormal expansion of these cells resulting in SOC. This is supported by observations in the fallopian tubes of human patients with the germline mutations in *BRCA1/2* genes that the earliest SOC precursor lesions are clonal expansions of few secretory cells [10]. In future studies, we plan to investigate the role of the fallopian tube stem/progenitor cells in the pathogenesis of ovarian cancer.

In summary, our examination of the fallopian tubes from women with hereditary predisposition to development of ovarian cancer revealed hyper activation of Wnt signalling in SOC precursor lesions. We developed a unique mouse model in which constitutive activation of βcatenin in the fallopian tube secretory cells causes development of similar precursor lesions confirming the involvement of deregulated Wnt/βcatenin involved in the initiation of SOC. Oestrogen treatment enhances and progesterone treatment suppresses tumorous growth in this mouse model by affecting Wnt/βcatenin signalling.

## MATERIALS AND METHODS

### Human fallopian tube tissue samples

This study is approved by the Institutional Human Research Ethics Committee at the University of Newcastle. Whole Fallopian tubes and ovaries were collected from the women who underwent risk reducing bilateral salpingo-oophorectomy at the John Hunter Hospital. Fallopian tube tissue samples were obtained from 17 patients. Patient information is presented in Table 1. Paraffin tissue blocks (10-16 blocks per high risk patient and 2-3 blocks per normal healthy woman) were sectioned at 6um thickness and 20 serial tissue sections were collected from every tissue block. The tissue sections encompassing the proximal, the middle and the distal ends of fallopian tubes were used for immunohistochemical marker analysis.

### Mouse genetics and husbandry

Mice used in the present study were maintained in standard animal housing conditions. The standard procedures and treatments performed on mouse models were approved by the Institutional Animal Care and Ethics Committee at the University of Newcastle. Pax8rtTA mice [40] were mated with tetOCre mice [41] to generate Pax8rtTa; tetOCre mice and referred to as LC1cre. LC1cre mice were crossed with Ctnnb1^fl(ex3)/fl(ex3^*^)^* mice [42] to develop LC1cre; Ctnnb1^fl(ex3)/fl(ex3)^ (βcatenin^ex3^cko). To generate lacZ reporter mice (LC1cre;lacZ^fl/+^), LC1cre mice were bred with Gt(ROSA)26Sor^tm1Joe^ (lacZ^fl/fl^) [43]. To induce recombination of flox alleles in βcatenin^ex3^cko and LC1cre;lacZ^fl/+^ mice, doxycycline (0.2-1mg/ml) was administrated in drinking water. Ear punch tissues were collected and genotyping PCRs were performed using REDExtract-N-Amp™ Tissue PCR Kit (Sigma, MO, USA). Recombination PCR to detect the floxed allele were done on DNA isolated from the fallopian tubes, ovaries and tails of βcatenin^ex3^cko mice. Primer details are listed in Table 2.

**Table 2:** List of primer pairs used for PCR

Transgene	Forward Primer	Reverse Primer
*tetOcre*	5′GCGGTCTGGCAGTAAAAACTATC3′	5′GTGAAACAGCATTGCTGTCACTT3′
*Pax8rtta*	5′CCATGTCTAGACTGGACAAGA3′	5′CTCCAGGCCACATATGATTAG3′
*Ctnnb1^F(lex3)^*	5′GACACCGCTGCGTGGACAATGA3′	5′GTGGCTGACAGCAGCTTTTCTA3′
*(ROSA)26Sor^tm1Joe^*	5′AAA GTC GCT CTG AGT TGT TAT3′ 5′TCC AGT TCA ACA TCA GCC GCT ACA3′	5′TAA GCC TGC CCA GAA GAC TC3′
*Ctnnb1^Fl(ex3)^* (Recombined allele)	5′- GGTAGGTGAAGCTCAGCGCAGAGC-3′	5′- ACGTGTGGCAAGTTCCGCGTCATCC-3′

### βgalactosidase staining

βgalactosidase staining was performed as described by us in [44]. Briefly, female reproductive tracts were collected from adult LC1cre;lacZ^fl/+^ mice and were fixed in 4% paraformaldehyde for 1hr at 4°C. Tissue were then washed in rinse buffer (0.1% sodium deoxycholate, 0.2% NP40, 2mM magnesium chloride in 0.1M phosphate buffer pH 7.3) for 30 min 3 times and stained with X-gal solution for 3-4 h at room temperature. Tissues were rinsed with PBS to remove excess of the solution and pictures were taken using Nikon SMZ25 stereoscope.

### Hormonal treatments

βcatenin^ex3^cko mice were treated with doxycycline for 4wks. These mutant mice were then ovariectomised and allowed to rest for 14 days to remove the traces of circulating hormones. 90-day slow releasing hormone pellets of 17-β-oestradiol (0.72 mg per pellet) or 17-β-oestradiol and Progesterone (0.72mg and 100mg per pellet; Innovative Research of America, Fl, USA) were subcutaneously inserted in mutant mice. Mutant mice with sham surgeries without any treatment were used as controls. After 46 days post-hormonal treatment, some of these mice presented with abnormal enlargement of peritoneum and all the mice were euthanized. The fallopian tubes were removed and fixed in 4% paraformaldehyde overnight at 4C.

### Histology, immunohistochemistry (IHC) and immunofluorescence (IF)

Hematoxylin and Eosin (H&E) staining was carried out using a standard protocol. IHC and IF protocols are described in [44]. The primary antibodies used in this study are as follows: βcatenin (1:200; BD Biosciences, NJ, USA), Cyclin d1 (1:100), LEF1 (1:100), Stathmin 1 (1:1200), TCF1 (1:100; Cell Signalling Technologies, MA, USA), ERα (1:500), PR (1:200; Santa Cruz Biotechnology, CA, USA), βgal (1: 500, MP Biomedicals, CA, USA), Ki67 (ready to use; Biogenex, CA, USA), Pax8 (1:500, Proteintech, IL, USA). AlexaFluor (Jackson ImmunoResearch Labs, PA, USA) or biotinylated (Biogenex) secondary antibodies were used. Stained slides were imaged at high resolution with the Olympus DP72 microscope or the Aperio Scanscope slide scanner. The gain and exposure time were set constant across tissue samples. Analysis for intensity and number of cells was done using the Halo Image analysis software (Indica labs, NM, USA).

### Western blotting

PE-01 cells were grown in 6-well plates and were treated with 17-β-oestradiol (500nM; Sigma, MO, USA)/ MPA (500nM; Sigma, MO, USA) or 17-β-estradiol (500nM) + MPA (500nM). Cells treated with DMSO were used as control. Protein was extracted using RIPA buffer. Equal amount of protein was loaded and β-actin was used as loading control. Primary antibodies: βcatenin (1:500; Cell Signalling Technology) and βactin (1:2000, Developmental Studies Hybridoma Bank, IA, USA). HRP conjugated secondary antibodies against mouse and rabbit were from Cell Signalling Technology or Jackson ImmunoResearch Laboratories. The mean density of the protein bands was determined using NIH Image J plugin.

### Cell culture and treatments

COV362 and PE01, SOC cell lines [45], were cultured in DMEM and RPMI-1640 added with 10% Foetal Bovine Serum (FBS), respectively. 5000 Cells were seeded per well in 96 well plates and treated with 17-β-Oestradiol, (10nM, 100nM and 500nM) or medroxyprogesterone acetate (MPA; 100nM, 250nM and 500nM) or DMSO for 72 hours. For carboplatin and MPA experiments, PE01 cells were plated in 96 well plates and following day these cells were subjected to one of the following treatments: MPA (250nM, 500nM) or MPA (250nM) plus carboplatin (10nM/25nM; Hospira, Pfizer, Australia). All drugs were replenished every 24 hours and the cells were assessed for cellular viability using Vision Blue Cell Viability Fluorometric Assay kit (Biovision, CA, USA). These experiments were repeated thrice.

### Statistical analysis

Graph Pad Prism 6.0 was used to conduct statistical analysis. All indicated values are in Mean ±SD and were subjected to one way ANOVA to assess differences between different groups with a *P* value less than 0.05 was considered statistically significant.

## SUPPLEMENTARY MATERIAL FIGURES


