# Supplementary material for: Ovarian hormones through Wnt signalling regulate the growth of human and mouse ovarian cancer initiating lesions
Source: Oncotarget. 2016 Aug 30;7(40):64836–53. doi: 10.18632/oncotarget.11711 (PMC5323120; doi:10.18632/oncotarget.11711)
Supplement: Supplementary file 1 [file oncotarget-07-64836-s001.pdf]

Ovarian hormones through Wnt signalling regulate the growth of human and mouse ovarian cancer initiating lesions

Supplementary Material

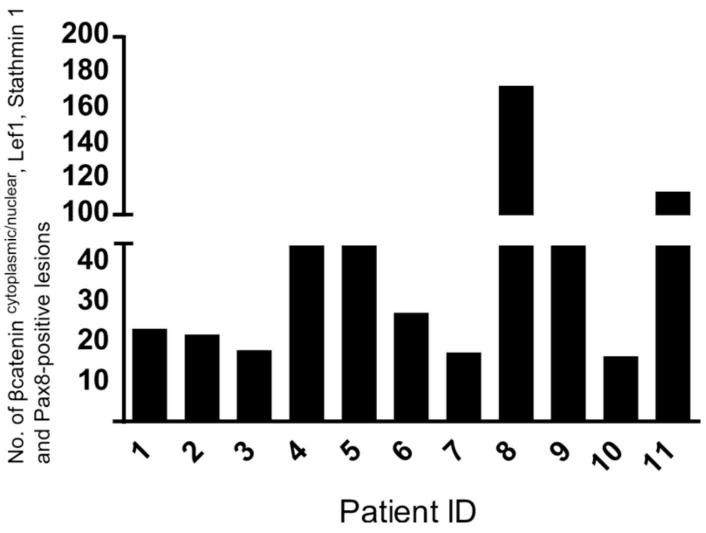

SFigure 1. Assessment of the βcatenin$^{nuclear/cytoplasmic}$, LEF1, Stathmin 1 and Pax8-positive ovarian cancer precursor lesions in the fallopian tubes collected from human patients with a hereditary predisposition to ovarian cancer development.

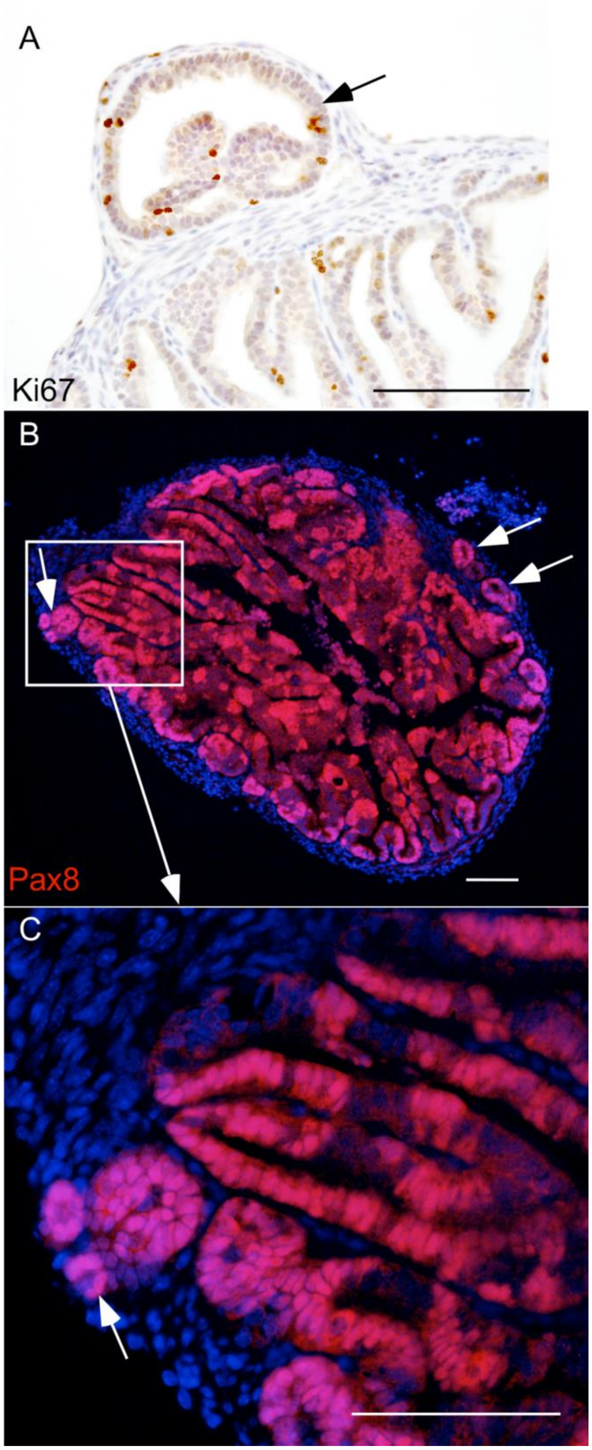

SFigure 2. Ki-67 staining showing invasive growth of the proliferating cells (arrow) in the serosal layer of oestrogen-treated mutant (bcatenin^{ex3} cko) oviducts (A). Pax8 staining of oestrogen-treated bcatenin^{ex3} cko oviducts (B). White arrows in panel B and C show Pax8-positive epithelial outgrowths in the serosal layer.
